# The microbial metabolite urolithin A reduces *Clostridioides difficile* toxin expression and toxin-induced epithelial damage

**DOI:** 10.1128/msystems.01255-23

**Published:** 2024-01-09

**Authors:** Sweta Ghosh, Daniel Erickson, Michelle J. Chua, James Collins, Venkatakrishna Rao Jala

**Affiliations:** 1 Department of Microbiology and Immunology, University of Louisville, Louisville, Kentucky, USA; 2 UofL-Brown Cancer Center, Louisville, Kentucky, USA; 3 Center for Predictive Medicine, University of Louisville, Louisville, Kentucky, USA; 4 Center for Microbiomics, Inflammation and Pathogenicity, University of Louisville, Louisville, Kentucky, USA; 5 Center for Integrative Environmental Health Sciences, University of Louisville, Louisville, Kentucky, USA; Johns Hopkins Bloomberg School of Public Health, Baltimore, Maryland, USA

**Keywords:** *Clostridioides difficile*, urolithin A, toxin production, gut barrier function, colitis

## Abstract

*Clostridioides difficile* is a Gram-positive, anaerobic, spore-forming bacterium responsible for antibiotic-associated pseudomembranous colitis. *Clostridioides difficile* infection (CDI) symptoms can range from diarrhea to life-threatening colon damage. Toxins produced by *C. difficile* (TcdA and TcdB) cause intestinal epithelial injury and lead to severe gut barrier dysfunction, stem cell damage, and impaired regeneration of the gut epithelium. Current treatment options for intestinal repair are limited. In this study, we demonstrate that treatment with the microbial metabolite urolithin A (UroA) attenuates CDI-induced adverse effects on the colon epithelium in a preclinical model of CDI-induced colitis. Moreover, our analysis suggests that UroA treatment protects against *C. difficile-*induced inflammation, disruption of gut barrier integrity, and intestinal tight junction proteins in the colon of CDI mice. Importantly, UroA treatment significantly reduced the expression and release of toxins from *C. difficile* without inducing bacterial cell death. These results indicate the direct regulatory effects of UroA on bacterial gene regulation. Overall, our findings reveal a novel aspect of UroA activity, as it appears to act at both the bacterial and host levels to protect against CDI-induced colitis pathogenesis. This research sheds light on a promising avenue for the development of novel treatments for *C. difficile* infection.

**IMPORTANCE:**

Therapy for *Clostridioides difficile* infections includes the use of antibiotics, immunosuppressors, and fecal microbiota transplantation. However, these treatments have several drawbacks, including the loss of colonization resistance, the promotion of autoimmune disorders, and the potential for unknown pathogens in donor samples. To date, the potential benefits of microbial metabolites in CDI-induced colitis have not been fully investigated. Here, we report for the first time that the microbial metabolite urolithin A has the potential to block toxin production from *C. difficile* and enhance gut barrier function to mitigate CDI-induced colitis.

## INTRODUCTION

To cause disease, *Clostridioides difficile* must overcome colonization resistance and invade the host microbiota. Despite successful antibiotic treatment for CDI, recovery of a functional intestinal epithelial barrier in patients with CDI is impaired. The *C. difficile* toxins TcdA and TcdB are principally responsible for damage to colonic epithelial and stem cells, respectively, triggering inflammation and impairing tight junction barrier function through Rho inhibition (1–4). Together, these can result in significant damage to the gut epithelium, leading to increased permeability, severe diarrhea, and colitis (5–7). Currently, no therapeutics are available for the treatment of *C. difficile*-induced gut barrier dysfunction or the promotion of functional intestinal epithelial barrier recovery following infection. Furthermore, current antibiotic therapies disrupt the commensal microbiota, resulting in the loss of colonization resistance against *C. difficile* and altering the balance of microbial metabolites with unknown implications.

Our group and others have investigated the mechanisms and benefits of the dietary microbial metabolite urolithin A (UroA, 3,8-dihydroxy-6H-benzo[c]chromen-6-one) under several pathophysiological conditions (8–14). UroA belongs to the family of urolithins and is characterized by a chemical structure containing an α-benzo-coumarin scaffold. Urolithins are produced in the gut following the microbiome-mediated transformation of the natural polyphenols ellagitannins (ETs) and ellagic acid (EA), which are present in dietary products, such as pomegranates, berries, and walnuts (15–25). Recently, *in vitro* culture studies have shown that *Bifidobacterium pseudocatenulatum* INIA P815 (26), *Enterococcus faecium* FUA027 (27), and *Streptococcus thermophilus* FUA329 (28) can metabolize ellagic acid to produce UroA. However, their role in UroA production *in vivo* has not yet been established. Studies indicate that only 40%–50% of people can produce UroA upon consuming these foods. This difference is attributed to the presence or absence of a specific bacterium or group of bacteria responsible for UroA production. When present, UroA can reach micromolar levels without toxicity in humans or mice (15, 29–33). UroA has recently been shown to reduce inflammation, enhance gut barrier function, and attenuate colitis in murine models in an aryl hydrocarbon receptor (AHR)-dependent manner (8, 29). As *C. difficile* is affected by intestinal metabolites (e.g., bile salts) and can shape its intestinal environment by triggering inflammation, gut barrier dysfunction, and increased gut permeability, we investigated whether UroA treatment could alleviate CDI pathogenesis.

## RESULTS

### UroA supplementation reduces CDI pathogenesis

**Fig 1:**
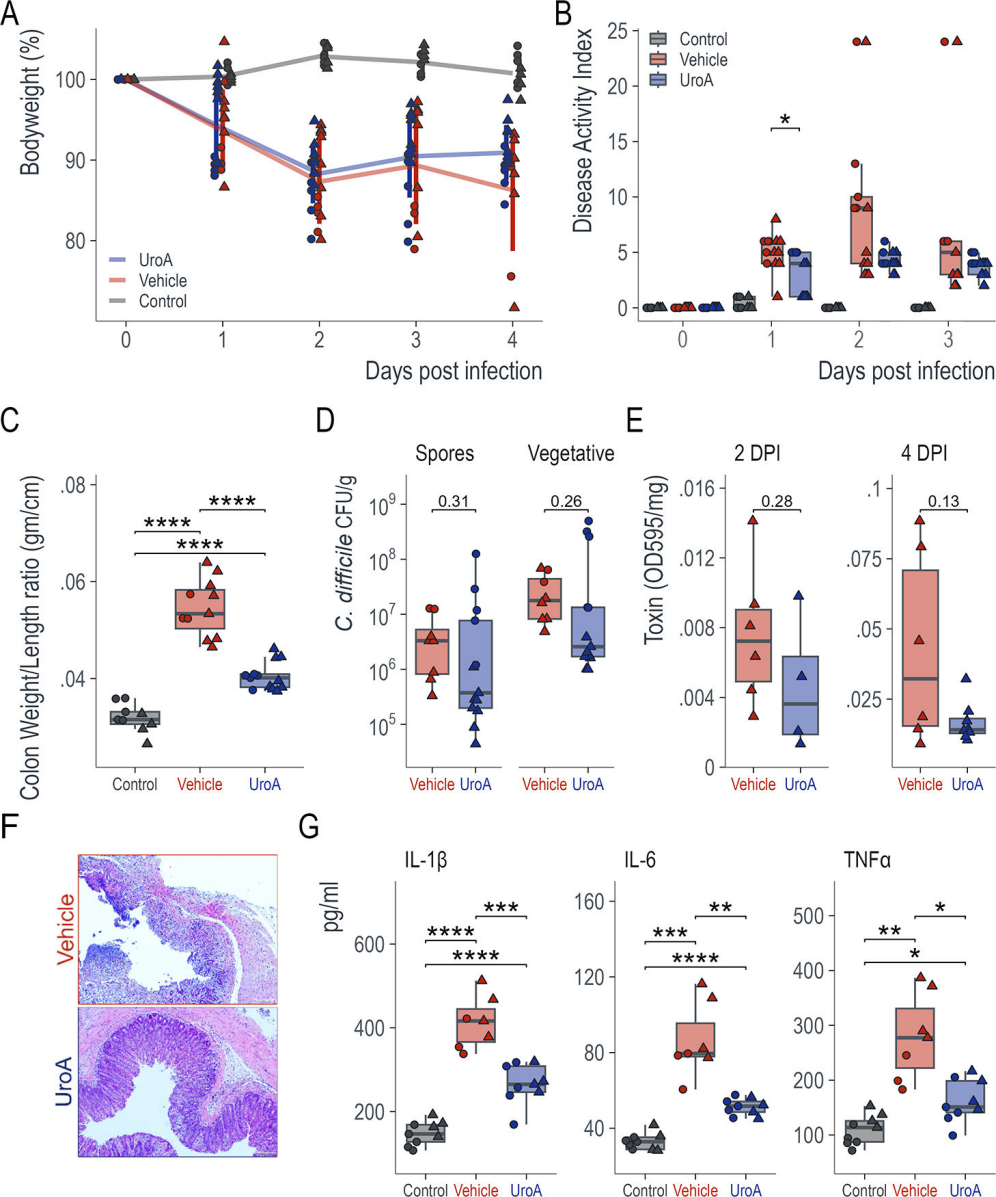
Urolithin A treatment reduces *C. difficile* pathogenesis. C57BL/6J mice (8 weeks old) were subjected to *C. difficile*-induced colitis. Two independent experiments were carried out, and the data were combined. Mice were divided into three groups: (i) control, antibiotics only (*n* = 9), (ii) *C. difficile* + vehicle (*n* = 13), and (iii) *C. difficile* + UroA (*n* = 13). (**A**) Percentage of body weight. Mice infected with *C. difficile* lost a significant amount of weight in both groups; however, 4 of the 13 mice (30.8%) in the vehicle group succumbed to infection, whereas all mice in the UroA group survived (*P* = 0.033, log-rank Mantel-Cox test). (**B**) DAI scores were given for all mice, with higher scores indicating more severe disease. The UroA group had significantly lower DAI scores than the vehicle group on day 1 (*P* = 0.01; Wilcoxon rank-sum test with continuity correction) and trended lower at all time points post-infection. (**C**) Colon length/weight ratio. Both the control and UroA groups had significantly lower length/weight ratios than the vehicle group (*P* < 0.0001 in both instances). (**D**) *C. difficile* burden. No significant difference in *the C. difficile* burden (spores or vegetative cells) was observed at 4 DPI (in stool). (**E**) Toxin levels trended lower at 2 DPI (from stool) and 4 DPI (from ceca). (**F**) H&E staining of colonic tissues from vehicle- and uroA-treated mice following *C. difficile* infection (representative images). (**G**) Mouse serum cytokine levels of IL-1β, IL-6, and TNF-α were measured using mouse-specific ELISA. Statistics in B, Wilcoxon test; D and E, Welch’s *t*-test; and C and G, one-way ANOVA with Tukey’s multiple comparison test. The circles and triangles represent data from Experiments A and B, respectively.

To determine whether UroA affects CDI severity, we challenged C57BL/6J mice with 10^6^ CD2015 spores (a clinical RT027 isolate). Mice were orally administered vehicle (1% CMC, 0.1% Tween 80) or UroA (20 mg/kg) on days 6, 5, 3, and 1 and daily from the day of infection. The mice were monitored daily for disease severity and euthanized on day 4 postinfection. Across two independent experiments, 4 of the 13 mice in the *C. difficile* +vehicle group died, whereas all mice (*n* = 13) in the *C. difficile* + UroA group survived (*P* = 0.033, log-rank Mantel-Cox test). Both the UroA and vehicle groups lost significant body weight compared with that in the control group (i.e., antibiotics only) (Fig. 1A). A standard clinical scoring system was used to evaluate the disease activity index (DAI) in the infected mice. The DAI scores reflected the phenotype, where the *C. difficile* + UroA group received significantly lower scores than the vehicle group on day one postinfection (*P* = 0.01; Wilcoxon rank-sum test with continuity correction; Fig. 1B) and trended lower throughout the experiment. An increase in the colon weight/length ratio is indicative of colonic inflammation. *C. difficile* infection caused a significant increase in colon weight/length ratio compared to control mice. However, treatment with UroA significantly ameliorated colonic inflammation in CDI mice (Fig. 1C). Despite the improvement in disease phenotype, the bacterial load (CFU) in the fecal samples did not show significant differences between the groups (Fig. 1D). Reduced toxin levels were observed in the UroA-treated mice on days 2 and 4 post-infection (from stool and ceca, respectively), although not significantly (Fig. 1E). Histopathological assessment of colon tissue showed that the UroA-treated mice had less damage and immune cell infiltration than the vehicle-treated mice (Fig. 1F). Furthermore, analysis of inflammatory cytokines in the serum suggested that UroA treatment downregulated *C. difficile*-induced increases in IL-1β, IL-6, and TNF-α levels (Fig. 1G).

### UroA treatment restores *C. difficile-*induced downregulation of tight junction proteins

**Fig 2:**
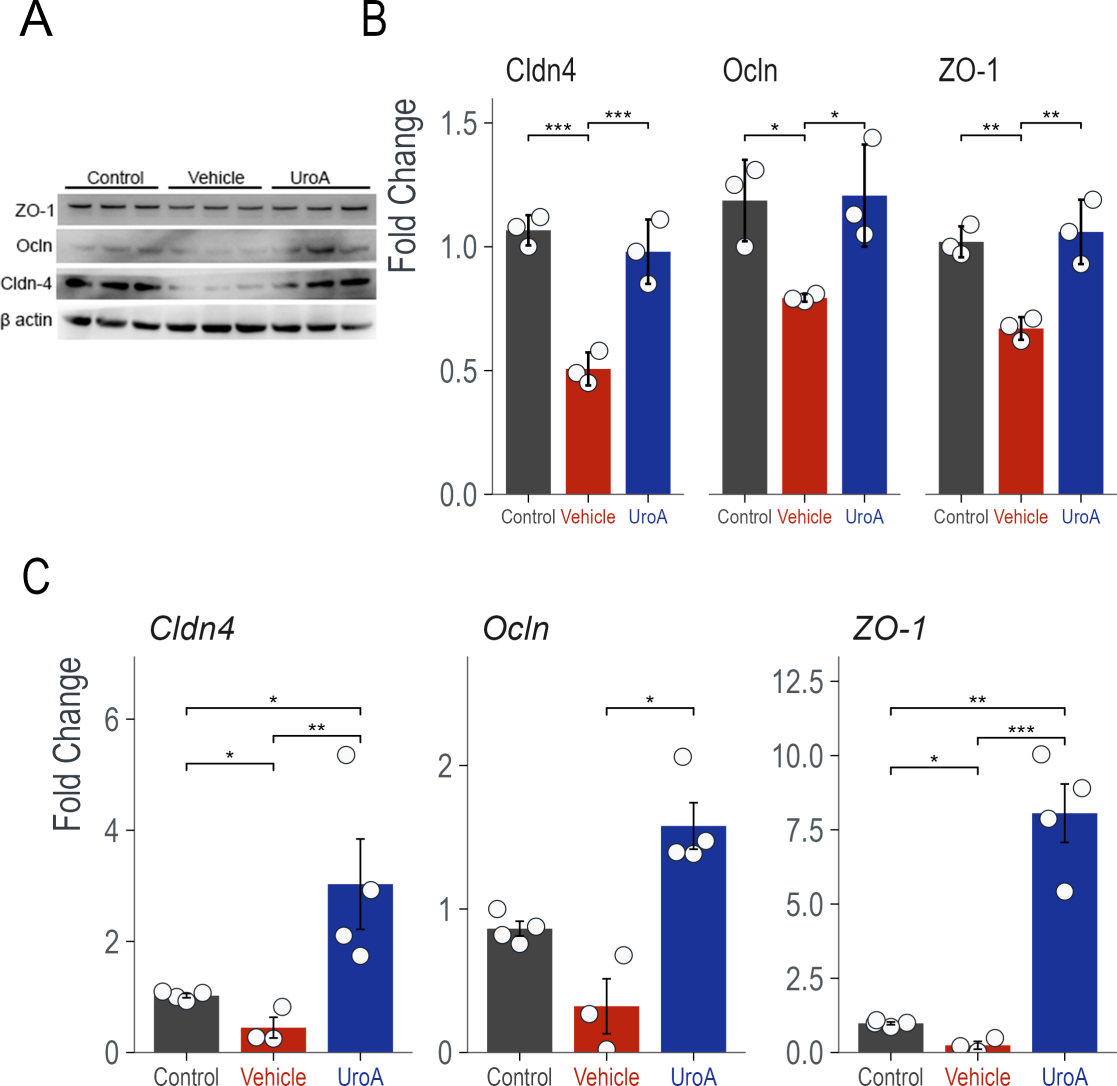
Urolithin A treatment protects CDI-induced tight junction protein (TJP) downregulation. *C. difficile* infection significantly reduces the TJPs claudin-4 (Cldn4), occludin (Ocln), and zonula occludens-1 (ZO-1). UroA treatment restores TJP levels. (**A**) Western blot of colonic TJPs. (**B**) Quantification of the western blots was performed using ImageJ software. (**C**) The mRNA levels of colonic TJPs were measured using qRT-PCR with SYBR Green. Statistics are performed using one-way ANOVA with Tukey’s multiple comparison test. *P < 0.05, **P < 0.01, and ***P < 0.001.

*C. difficile* toxins can disrupt tight junction proteins, contributing to enhanced paracellular permeability and disease pathophysiology of pseudomembranous colitis (34). Analysis of murine colons following infection showed that mice infected with *C. difficile* had significantly downregulated colon tight junction proteins (TJPs; ZO-1, OCLN, and CLDN4). However, UroA treatment protected and restored the TJPs at the protein level (Fig. 2A and B) and mRNA levels (Fig. 2C). These results suggest that UroA treatment reduces overall inflammation and protects against CDI-induced TJP disruption.

### UroA is not a bactericidal

**Fig 3:**
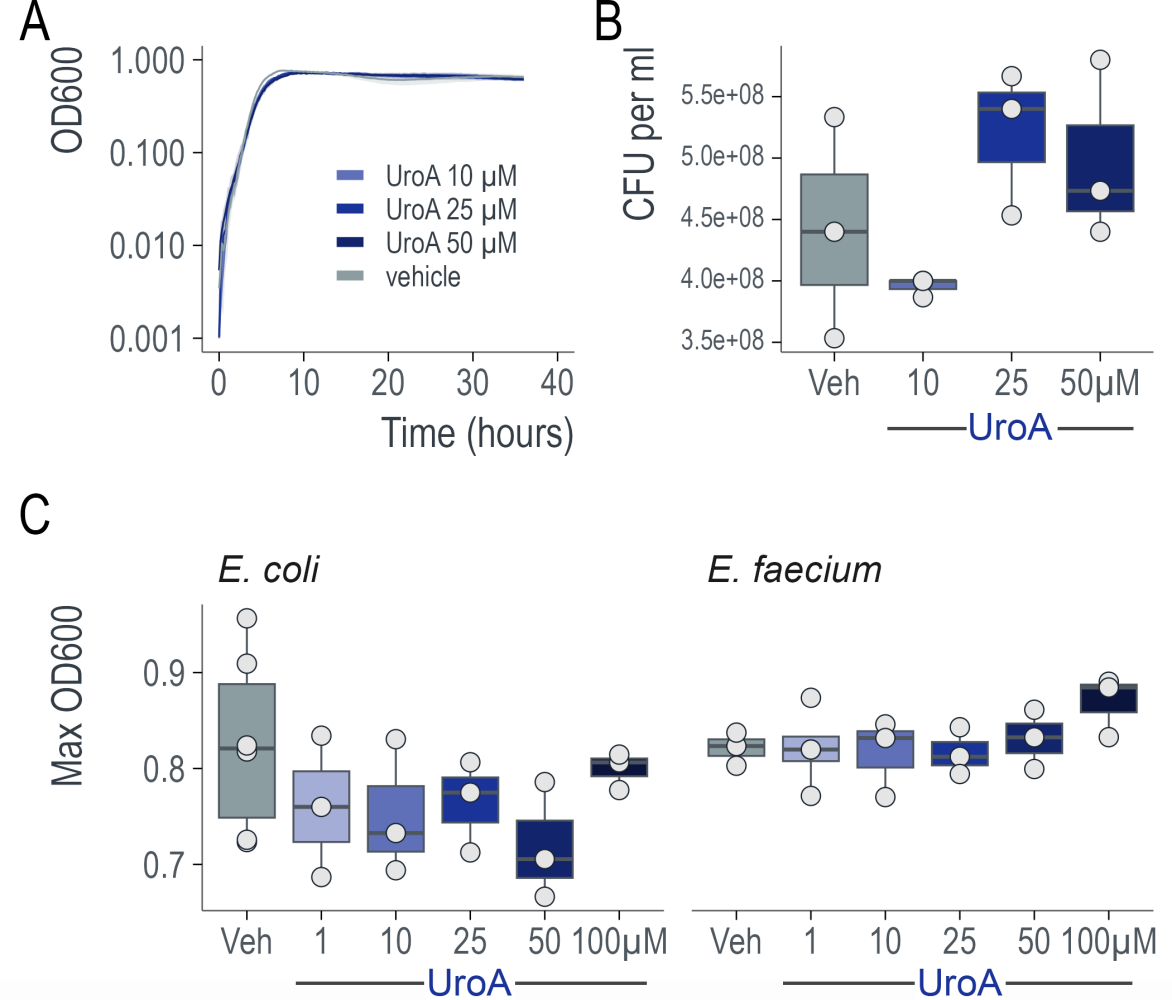
Urolithin A is neither bactericidal nor bacteriostatic. (**A**) UroA did not affect *C. difficile* growth. Three biological replicates of *C. difficile* CD2015 were grown in BHIS medium supplemented with 0–50 µM of UroA. No significant differences were observed in the growth rate or maximum OD. The curves represent mean ± SD. (**B**) UroA treatment did not affect *C. difficile* viability. After 36 h of growth in BHIS + 0–50 µM of UroA, cells were diluted and plated onto BHIS agar plates. No significant differences were observed in the number of viable cells (ANOVA). (**C**) UroA does not affect the growth of other common intestinal microbes. *E. coli* and *E. faecium* were grown in LB and BHI media, respectively, containing 0–100 µM of UroA. No difference in growth (max OD) was observed (ANOVA).

We observed no significant difference in *C. difficile* burden between the vehicle and UroA groups. This suggested that UroA does not act as an antibacterial agent against *C. difficile*. To confirm this observation and determine whether UroA elicited an antibacterial or bacteriostatic effect, we grew CD2015 in a BHI medium containing increasing concentrations (0–50 µM) of UroA for 36 h and measured the OD600 every 10 min. No significant differences in doubling time or maximum OD were observed under any growth condition (Fig. 3A). It is possible that nonviable stationary-phase cells produce the same OD600 values as viable cells. To test whether the OD600 values masked the true bacterial viability after 36 h of growth, the samples were diluted and plated on BHI plates without the spore germinant taurocholate to enumerate the viable vegetative *C. difficile*. No significant difference was observed in CFUs for any of the UroA concentrations (*P* = 0.164; ANOVA; Fig. 3B). To test for bactericidal effects more broadly, we grew the common gut commensals *Escherichia coli* and *Enterococcus faecium* in rich media containing 0–100 µM of UroA. Again, no significant differences in growth or maximal OD600 were observed (*P* = 0.394 and *P* = 0.387, respectively; ANOVA; Fig. 3C). These data implied that UroA does not exhibit bactericidal or bacteriostatic effects at physiological concentrations.

### UroA reduces toxin from *C. difficile* in a dose-dependent manner

**Fig 4:**
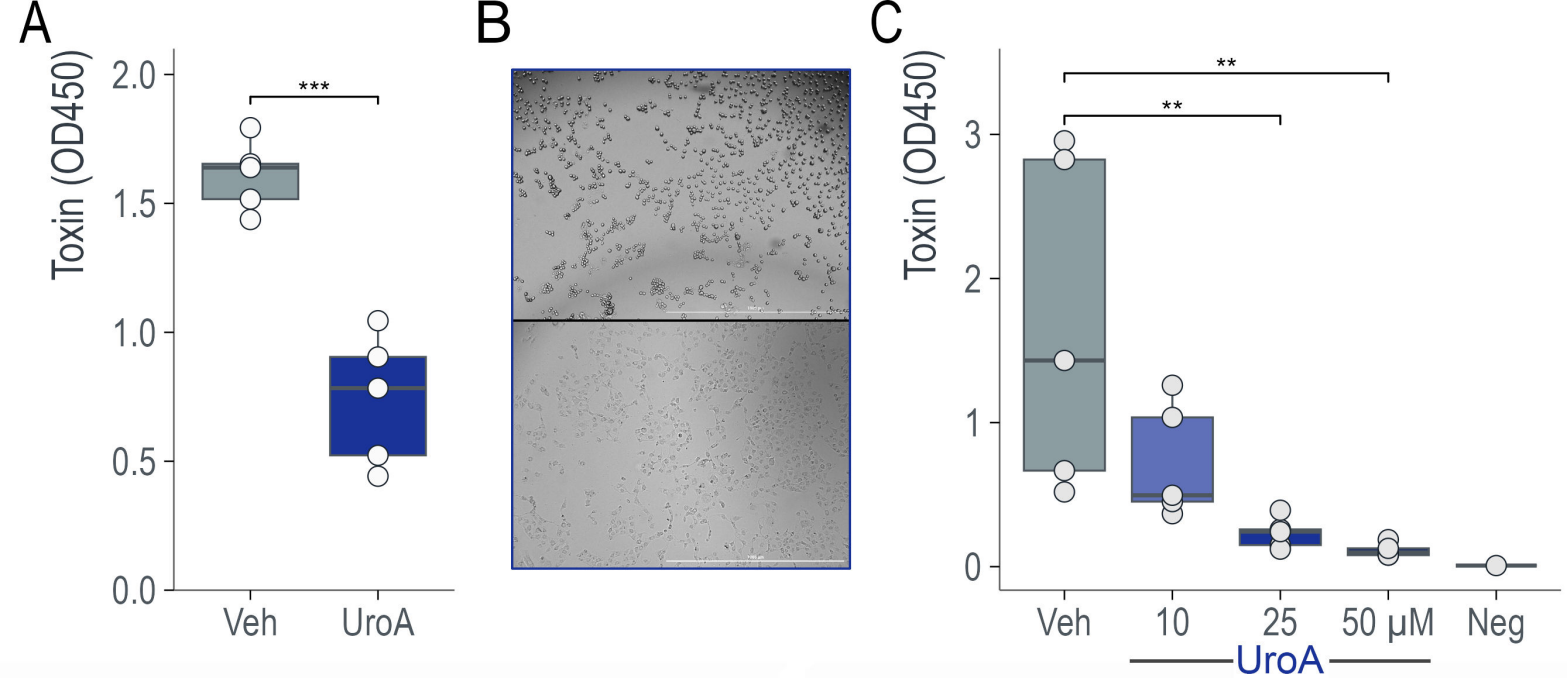
UroA reduced *C. difficile* toxin levels in a dose-dependent manner. (**A**) Five biological replicates of CD2015 were grown in a BHI medium supplemented with 25 µM UroA or the vehicle control for 24 h. UroA treatment significantly reduced toxin levels (*P* = 0.000151, TcdA, and TcdB, as measured by non-specific ELISA). (**B**) *C. difficile* was grown in 4 mL of BHIS media containing vehicle (0.1% DMSO) or UroA (50 µM; *n* = 6 per group) for 24 h. One millimeter of supernatants (sup) was collected, centrifuged, and filtered through a 0.2-µM filter. The supernatants were diluted in complete Vero-2 cell media (EMEM containing 10% FBS and 1% penicillin streptomycin). The Vero-2 cells (60%–80% confluence in a 96-well plate) were treated with diluted *C. difficile* supernatants (sup) with media at 50:50. Representative images of vehicle-treated *C. difficile* sup (top panel) and UroA-treated *C. difficile* sup (bottom panel) at 3 h posttreatment are shown. The scale bar indicates 1 mm. (**C**) Five biological replicates of CD2015 were grown in a BHI medium supplemented with 10–50 µM of UroA or vehicle control for 36 h. UroA treatment significantly reduced toxin levels in a dose-dependent manner, as measured by ELISA. ANOVA with *post hoc* Dunnett’s test.

To determine whether UroA affects toxin expression, *C. difficile* was grown in BHI medium with or without 25 µM UroA for 24 h, and the levels of toxins in the supernatants were measured using ELISA. Upon UroA treatment, the TcdA and TcdB protein levels were significantly reduced (Fig. 4A). To confirm the functional reduction in toxin levels, a Vero cell rounding assay was performed with the supernatant after 24 h of growth. Cells treated with the supernatant from the *C. difficile* + vehicle group exhibited a cell rounding phenotype, indicative of toxicity. In contrast, cells treated with the supernatant from the UroA-treated group failed to cause cell rounding (Fig. 4B). To determine whether the effect of UroA on *C. difficile* toxins was dose-dependent, we grew *C. difficile* in BHI medium containing increasing concentrations (0–50 µM) of UroA for 36 h and measured the toxins in the supernatant using ELISA. Figure 4C clearly shows the dose-dependent reduction in *C. difficile* toxin levels. These data, in combination with growth data, suggest that UroA directly interacts with *C. difficile* to downregulate toxin production in a growth-independent manner.

### UroA downregulates genes in the pathogenicity locus

**Fig 5:**
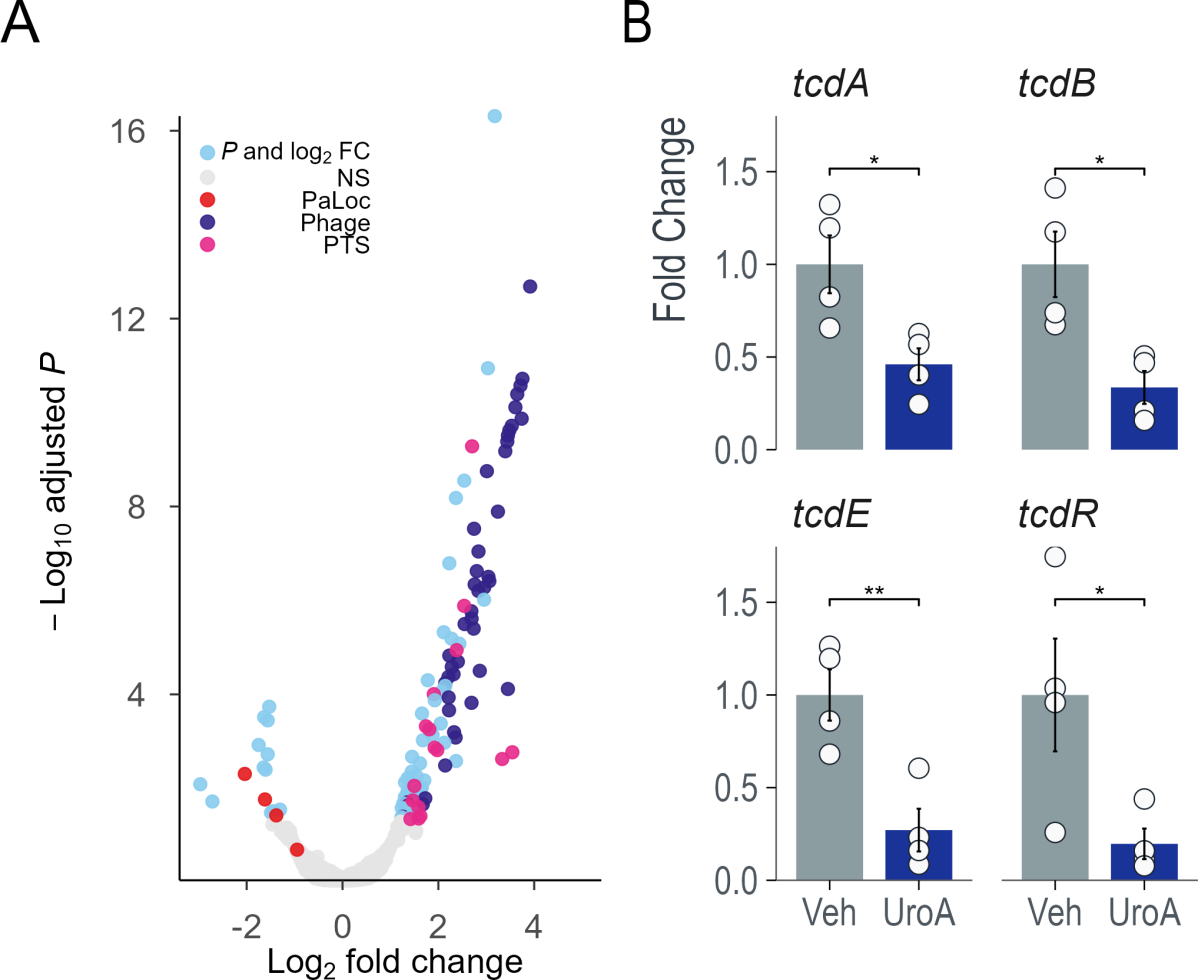
Urolithin A reduces toxin gene expression. Four biological replicates of CD2015 were grown in BHI ± 25 µM UroA or vehicle for 24 h. (**A**) RNA-Seq analysis revealed that PaLoc genes were downregulated in the UroA group, whereas several PTS and phage operons were significantly upregulated. (**B**) The main toxin genes, *tcdA* and *tcdB,* were downregulated, as were *tcdE* (encoding holin) and *tcdR* (a positive regulator of toxin gene expression).

*C. difficile* produces toxins during stationary-phase growth *in vitro*. To better understand the transcriptional landscape during this phase, we performed RNA-Seq on *C. difficile* grown in the presence or absence of 25 µM UroA for 24 h. In total, 109 genes were significantly upregulated, and 14 genes were downregulated in the presence of UroA (using a threshold of false discovery rate  <0.05 log_2_ fold change >1; Fig. 5A; Table S1). A significant portion (40%) of the upregulated genes were phage-associated from two large operons. Lysogenic phage often induces a lytic cycle when the host encounters unfavorable environmental conditions, though we did not observe any difference in viable vegetative cells. Three phosphotransferase (PTS) operons, accounting for 12 genes, were also upregulated. Several genes located in the pathogenicity locus (PaLoc) were downregulated, including *tcdA*, *tcdB*, *tcdE* [encoding a holin that mediates toxin release from *C. difficile* cells (35, 36)], and *tcdR* (Fig. 5B). TcdR is an alternate sigma factor that directs transcription by recruiting RNA polymerase to the toxin gene promoters and its own promoter (37, 38). These data suggest that UroA can downregulate toxin gene expression via TcdR, either directly or through an alternative mechanism; however, further research is required to elucidate the full mechanism.

## DISCUSSION

During active CDI, *C. difficile* induces host pathology and inflammation, which can further exacerbate microbial dysbiosis and decrease the beneficial microbial metabolites required for gut homeostasis. Several studies have highlighted the importance of microbial metabolites in the regulation of gut barrier function by regulating both the immune and epithelial systems (39–42).

Microbial metabolites, such as short-chain fatty acids (SCFAs), indoles, purines, secondary bile acids, and polyamines, play a role in maintaining and restoring gut barrier function and mucin production (39–42). Patients with CDI have decreased levels of tight junction proteins and increased gut barrier dysfunctions (43). Furthermore, those patients that go on to have recurring CDI exhibit an altered gut metabolome indicative of reduced gut microbiome function, host inflammation, and reduced immunomodulatory capabilities (44).

Several microbiota-derived compounds have been shown to modulate pathogen virulence factors. Of these, SCFAs are a well-studied class with an established role in the modulation of enteric infections by *Salmonella*, *Listeria*, *Campylobacter*, *Shigella*, and *E. coli* (45). In *C. difficile*, SCFAs have a direct inhibitory effect on growth with acetate, butyrate, propionate, and valerate, reducing the growth rate of *C. difficile* in culture (46–48). Indole, a microbial metabolite of tryptophan, reduces enterohemorrhagic *Escherichia coli* O157:H7 attachment to intestinal epithelial cells and biofilm formation and attenuates *Salmonella typhimurium* virulence and invasion, likely due to a decrease in the expression of multiple *Salmonella* pathogenicity island-1 (SPI-1) genes (49).

Toxins (TcdA and TcdB) from *C. difficile* cause epithelial damage in the intestines, leading to increased permeability and downregulation of tight junction proteins. Toxins A and B are glucosyltransferases that inactivate Rho and Ras family GTPases within target epithelial cells, resulting in loss of structural integrity and cell death by apoptosis. Both toxins can also induce pro-inflammatory signaling pathways in the host (50, 51). A recent study by Mileto et al. (4) delineated the toxic effects of TcdA and TcdB by using specific knockout strains. TcdA disrupts the integrity of the gut epithelial barrier, whereas TcdB damages colonic stem cells and impairs epithelial healing (4). Therefore, therapeutics that target toxin production and protect against TcdA- and TcdB-induced gut damage may mitigate CDI pathogenesis. Bezlotoxumab is an antitoxin B monoclonal antibody that reduces the likelihood of CDI recurrence, although the mechanism by which this risk is mitigated remains unclear (52).

In the gut, UroA has been shown to reduce colon injury severity, inflammation, and intestinal permeability while improving mucosal integrity in several models (8, 29). In this study, we tested the hypothesis that supplementation with UroA protects against CDI by enhancing gut barrier function through the upregulation of tight junction proteins. Previously, our group demonstrated that treatment with UroA enhances gut barrier function, reduces inflammation, and attenuates colitis in murine models in an AHR-dependent manner (8). We adopted a CDI preclinical model in which UroA supplementation significantly downregulated CDI pathogenesis (Fig. 1) including decreased DAI scores, reduced systemic inflammation, and mitigated CDI-induced shortening of colons. It was also evident from the H&E analysis of colon sections that UroA restored or inhibited CDI-induced gut epithelial damage (Fig. 1). The variations in overall toxin levels in fecal samples between mice administered with a vehicle or UroA did not attain statistical significance. Nevertheless, we did discern a declining trend in toxin levels among mice treated with UroA. It is possible that UroA treatment may reduce the toxin levels in the cecum or at the actual sites of *C. difficile* infection within the intestines. This hypothesis warrants further investigation. Importantly, UroA treatment restored CDI-induced downregulation of TJPs (Fig. 2).

Recent studies have highlighted that AHR activation in the intestine leads to stem cell activation, leading to the proliferation of intestinal epithelial cells (53, 54). We predict that activation of AHR by UroA may assist in the regeneration of the intestinal epithelium and induce tight junction proteins during CDI. In addition, we previously showed that UroA treatment protects the gut barrier from endotoxin- and TNF-α/IFN-γ-induced permeability and downregulation of tight junction proteins. It is also possible that UroA may reduce CDI-induced inflammatory cytokine-mediated gut barrier dysfunction. We postulate that UroA potentially regulates multiple levels to protect the host from adverse effects.

To the best of our knowledge, no studies have reported a direct effect of microbial metabolites on *C. difficile* virulence without affecting growth. Mahnic et al. (55) reported that bioreactors in which human-derived microbiota exposed to pomegranate polyphenol extract (predominantly ellagitannins that can be converted by gut microflora to urolithin A derivatives) in combination with clindamycin prior to *C. difficile* infection resulted in significantly less relative cytotoxicity units per *C. difficile* CFU (RCU/*C. difficile* CFU) than those in the other treatments. The microbiota used was pooled from two subjects, and it is unknown whether they could produce UroA. Therefore, the mechanism of toxin reduction may have involved the production of UroA; however, this was not explicitly tested.

To determine whether UroA exhibits antibacterial activity, we grew bacteria in the presence of UroA. Our data indicated that UroA did not show any bactericidal activity. However, to our surprise, UroA treatment significantly downregulated the secretion of toxins from *C. difficile* at both protein and mRNA levels. RNA-Seq revealed that UroA robustly downregulated several genes in the PaLoc, including toxin genes and *tcdR*, a toxin gene regulator. TcdR is a central regulator of *C. difficile* toxin production. Other proteins directly regulate tcdR transcription and toxins. For example, CodY, a global transcriptional regulator, represses toxin gene expression by binding to the *tcdR* promoter region with high affinity (56, 57). The sigma factor SigD positively regulates toxin production by controlling *tcdR* transcription (58). Finally, in response to sugar availability, CcpA, a major regulator of carbon catabolite repression, binds to the promoter region or 5′ ends of several PaLoc genes, with the strongest affinity for the promoter region of tcdR (59, 60). Several PTS operons were significantly upregulated in the presence of UroA, suggesting CcpA may play a role. Further studies are required to define the UroA target and regulatory mechanisms responsible for toxin expression in *C. difficile*. In conclusion, this study provides, for the first time, an insight into how the microbial metabolite UroA can interact with the host and *C. difficile* to reduce disease severity and promote intestinal healing.

## MATERIALS AND METHODS

### Bacterial strains and culture conditions

*C. difficile* CD2015 (clinical RT027 strain) was stored at −80°C in BHIS [BHI medium (Difco) supplemented with 0.5% (wt/vol) yeast extract ] + 10% DMSO. When required, CD2015 was struck onto BHIS plates, and single colonies were inoculated into liquid BHIS or BHI (for toxin assays) at 37°C under anaerobic conditions (5% hydrogen, 90% nitrogen, 5% carbon dioxide). *E. coli* and *E. faecium* were stored at −80°C in LB or BHI supplemented with 10% DMSO and grown aerobically in LB and BHI, respectively.

For growth curves, strains were grown in appropriate media overnight (BHIS, BHI, LB), back diluted to an OD600 of 0.01, and inoculated 1:10 into appropriate media containing UroA or DMSO vehicle control. Technical triplicates for each biological replicate were used in a sterile 96-well plate, and the OD600 was measured every 10 min using a Cerillo plate reader.

### Vero cell rounding assay

Vero cells (an African green monkey kidney cell line) were purchased from ATCC (CCL-81). 1 × 10^4^ cells per well were seeded in a 96-well plate. Cells were grown in Eagle’s minimum essential medium (EMEM), supplemented with 10% fetal bovine serum, and supplemented with 5 µg/mL penicillin and 5 µg/mL streptomycin sulfate. Cells were grown for 24 h at 37°C, 5% CO_2_ in air atmosphere reaching up to 60%–80% confluency. *C. difficile* was grown in 4 mL of BHIS media containing vehicle (0.1% DMSO) or UroA (50 µM; *n* = 6 per group) for 24 h. One milliliter of supernatants (sup) was collected, centrifuged, and filtered through a 0.2 micron filter. The supernatants were diluted in complete Vero-2 cell media. The Vero-2 cells (60%–80% confluence in a 96-well plate) were treated with different dilutions of *C. difficile* supernatants (sup) with media. The images were collected 3 h posttreatment by BioTek Cytation 5 cell imaging station.

### Mouse model of *C. difficile* infection

Mice were administered a five-antibiotic cocktail as described in Collins et al. (61). Briefly, kanamycin (0.4 mg mL^−1^), gentamicin (0.035 mg mL^−1^), colistin (850 U mL^−1^), metronidazole (0.215 mg mL^−1^), and vancomycin (0.045 mg mL^−1^) were administered *ad libitum* in drinking water for 3 days. The water was switched to antibiotic-free sterile water, and 24 h later, the mice were administered an intraperitoneal injection of clindamycin [10 mg per kg (body weight)]. After 24 h (day 0), the mice were challenged with 10^6^ *C. difficile* spores via oral gavage. Mice were orally administered vehicle (1% CMC, 0.1% Tween 80) or UroA (20 mg/kg) on days 6, 5, 3, and 1 and daily from the day of infection. Mice were euthanized on day 4 post-infection and characterized colitis phenotype.

### Evaluation of DAI

Body weight and DAI were recorded daily. The DAI was determined by combining the scores for (i) activity, (ii) posture, (iii) coat, (iv) stool consistency, (v) eyes/nose, and (vi) body weight. Scores for i–v ranged from 0 (normal) to 3 (most severe) in each area and were scored by a blinded researcher. Body weight loss was calculated as the percentage of the difference between the original body weight (day 0) and the body weight on any day and scored as per Shelby et al. (62). Briefly, mice weighing 96%–99%, 91%–95%, 86%–90%, and ≤85% of their original weight scored 1, 2, 3, or 4 points, respectively. At the end of the experiment, all mice were sacrificed, and the large intestines were separated from the vermiform appendix to the anus. Colon length was measured between the cecum and proximal rectum.

### RNA sequencing

Four independent biological replicates of *C. difficile* CD2015 were grown in BHI medium supplemented with 25 µM UroA or vehicle (DMSO) for 24 h. Following growth, cells were pelleted, supernatant was removed, 1 mL of RNA later was added, and the pellet was stored at −80°C until needed. Total RNA was extracted using the RNeasy kit (Qiagen), and residual DNA was removed using the TURBO DNA-free Kit (Invitrogen). RNA-Seq was performed at SeqCoast Genomics. RNA samples were subjected to ribosome depletion and sequenced on an Illumina NextSeq 2000 platform using a 300-cycle flow cell kit to produce 2 × 150-bp paired reads. After demultiplexing, read trimming, and FastQC analysis, transcript expression was determined using Salmon in Python 3.10 and subsequent analysis with DESeq2 and apeglm in R v4.3.0 (63–66).

### ELISA

Toxins were measured with the fecal *C. difficile* toxin AB qualitative ELISA assay kit (Eagle Biosciences). Cells were removed from the supernatant via centrifugation, followed by filtration through a 0.22-µM filter. The supernatants were diluted 1:5 in PBS before following the kit instructions.

### Western blot analysis

For western blot analysis, colon tissues were homogenized and lysed using RIPA buffer containing 1× protease inhibitors (Sigma-Aldrich, MO, USA), and lysates were further processed for immunoblotting as previously described (67). The membranes were probed with ZO-1, occludin, Cldn-4, and β-actin antibodies, followed by incubation with a secondary antibody conjugated with horseradish peroxidase (Proteintech, IL, USA). The protein bands were developed with Immobilon Forte Western HRP substrate (Millipore Sigma, MA, USA) and imaged using a Bio-Rad ChemiDoc Imaging System (Hercules, CA, USA). Densitometric analysis of the bands was performed using ImageJ software (68). A list of the antibodies, sources, and dilutions used is provided in Table S2.

### Real-time quantitative polymerase chain reaction (RT-qPCR)

Total RNA from colon tissues was isolated using Maxwell 16 LEV simplyRNA tissue kits (Promega, WI, USA) following the manufacturer’s instructions. Changes in the expression of ZO-1, occludin, and Cldn4 genes were evaluated as described previously (9). Fold changes in gene expression were estimated using the ^-ΔΔ^CT method, with β-actin as a housekeeping gene control and normalized to the control.

### Measurements of serum cytokines

Mouse serum cytokine levels were measured for TNF-α, IL-6, and IL-1β using mouse-specific ELISA kits (BioLegend, CA, USA) following the manufacturer’s instructions.

### Histopathology and immunohistochemistry of colon tissue

Mouse colons were fixed in a 10% buffered formaldehyde solution overnight, followed by a 70% alcohol change. Fixed tissues were subjected to standard histopathological processing for paraffin embedding, and 5 µM paraffin sections were cut and stained with hematoxylin and eosin (H&E) by Saffron Scientific Histology Services (IL, USA).

### Statistical analysis

Statistical analysis was performed using R v4.3 (63). Details of the specific tests can be found in the relevant figure legends: *P < 0.05, **P < 0.01, and ***P < 0.001. Fold change values were normalized via log_2_ transformation prior to statistical analysis.

## Data Availability

Data on the normalized transcript abundance and differential analyses are shown in Table S1. Prior to publication of the peer-reviewed manuscript, raw data from the RNA-Seq experiment shown in Fig. 5 will be available from the corresponding author upon request. Raw data will be uploaded to NCBI and made freely available upon acceptance of the peer-reviewed manuscript.

## SUPPLEMENTAL MATERIAL

Table S1 msystems.01255-23-s0001.xlsx UroA-induced genes in *C. difficile*.

Table S2 msystems.01255-23-s0002.xlsx List of antibodies.

ASM does not own the copyrights to Supplemental Material that may be linked to, or accessed through, an article. The authors have granted ASM a non-exclusive, world-wide license to publish the Supplemental Material files. Please contact the corresponding author directly for reuse.
